# Ultrasound as a replacement for physical examination in clinical staging of axillary lymph nodes in breast cancer patients

**DOI:** 10.1111/1759-7714.13224

**Published:** 2019-11-11

**Authors:** Xue Chen, Xiaoting Li, Zhaoqing Fan, Jinfeng Li, Yuntao Xie, Tianfeng Wang, Tao Ouyang

**Affiliations:** ^1^ Breast Cancer Prevention & Treatment Center, Key Laboratory of Carcinogenesis and Translational Research (Ministry of Education) Peking University Cancer Hospital & Institute Beijing China; ^2^ Department of Radiology, Key Laboratory of Carcinogenesis and Translational Research (Ministry of Education) Peking University Cancer Hospital & Institute Beijing China

**Keywords:** Axillary lymph nodes staging, breast cancer, physical examination, ultrasound

## Abstract

**Background:**

The status of axillary lymph nodes (ALNs) is one of the important factors in decision‐making for breast cancer treatment. Physical examination (PE) has long been the main, or even the only, means of clinical staging for ALNs in breast cancer. However, the sensitivity and accuracy of PE remains unsatisfactory. The results from this study suggest that axillary ultrasonography (US) should replace PE as a standard method for the clinical staging of ALNs in breast cancer.

**Methods:**

Consecutive and nonselective breast cancer patients treated between September 2018 and November 2018 in our center were enrolled in the study. Comparisons of ALN results between PE/US and pathological results were conducted and the difference in sensitivity, specificity and accuracy between PE and US were tested by McNemar chi‐square test.

**Results:**

A total of 123 patients were enrolled into the study. Their ages ranged from 28 to 76 years with a median age of 53 ± 10. There were 83 ALN positive cases and 40 ALN negative cases confirmed pathologically. The sensitivity, specificity, accuracy, positive predictive value and negative predictive value of PE and US were 54.2%, 90.0%, 65.9%, 91.8%, 48.7% versus 86.8%, 72.5%, 82.1%, 86.8%, 72.5%, respectively. The sensitivity and accuracy of US was significantly higher than that of PE (*P* = 0.004 and *P* = 0.002).

**Conclusion:**

The results of this study demonstrated that US is superior in evaluating ALNs when compared with PE and that US should replace PE as the standard method for the clinical staging of ALNs in breast cancer.

## Introduction

The pathological status of axillary lymph nodes (ALNs) is one of the important factors in decision‐making of the treatment for breast cancer. The accurate clinical staging of ALNs is the premise for whether to choose an individualized minimally invasive biopsy and the guarantee of precise acquisition of ALN tissue. Unfortunately, for a long time, in most centers, subjective physical examination (PE) is still the main, or even the only, means of ALN clinical staging in breast cancer management. Due to its strong subjectivity, poor objective repeatability of the results, as well as different training level and experience of PE physicians, the outcome of PE is influenced by many factors, with its accuracy also greatly questioned.^1^ Therefore, it is impossible to decide whether to choose the ALN biopsy method or ultrasound‐guided core needle biopsy (CNB) based on the results of PE.

However, modern objective methods such as mammography, computed tomography (CT), positron emission tomography (PET)‐CT and magnetic resonance imaging (MRI) have their own limitations because of the diversity of the breast itself which is different from other organs.^1^ So far, these examinations have not been listed as the routine means of clinical staging for ALNs in breast cancer.^1^ Ultrasound (US) is a noninvasive and convenient objective examination, which not only shows high sensitivity, specificity, accuracy and repeatability in the clinical staging of ALNs in breast cancer,^2^, ^3^, ^4^ but also allows for dynamic, timely and multidirection evaluation with differentially individual anatomical and physiological characteristics of the breast and axilla, which to some extent retains major advantages of PE. More importantly, the minimally invasive biopsy of ALNs can be individualized by US according to the different performance of ALNs, and the accuracy of the biopsy can be precisely guided by US. Therefore, US has attracted more and more attention in the clinical staging of ALNs in breast cancer. Particularly, in high‐volume breast cancer centers, there is a tendency to gradually replace the traditional subjective PE by the US results from specialist US physicians.

However, limited data is available regarding the comparison between the subjective PE and the objective US in the clinical staging of ALNs in breast cancer. Therefore, whether US is superior to PE in the clinical staging of ALNs in breast cancer and whether US should replace PE as the only clinical staging method of ALNs still remains controversial.

## Methods

A total of 222 consecutive and nonselective breast cancer patients were treated between September 2018 and November 2018 at the Breast Cancer Center of Peking University Cancer Hospital. Informed consent was obtained from all individual participants included in the study.

### Inclusion criteria

Primary invasive breast cancer diagnosed by CNB, and patients who had not received any neoadjuvant therapy were included in the study.

### Exclusion criteria

Patients were excluded from the study for the following reasons: (i) Carcinoma in situ as diagnosed by CNB; (ii) primary invasive breast cancer diagnosed by resection; (iii) patients who had received neoadjuvant therapy; (iv) the pathology of sentinel lymph node biopsy (SLNB) showed micrometastasis or isolated tumor cells (ITC); (v) T4 patients; (vi) local recurrence cases after treatment; (vii) patients who had rejected SLNB; (viii) failure of SLNB and (ix) patients who ceased treatment due to personal reasons. Both PE and US was performed for ALNs in all 222 cases.

### Physical examination of ALNs

The PE of ALNs was performed by senior specialists who had completed a full training programme in our center. For those patients with no palpable enlarged ALNs, cN0 was recorded; for those with palpable enlarged ALNs, cN1 was recorded.

### Ultrasound examination of ALNs

The US examination of ALNs was performed by specialist breast ultrasound physicians from our center. The result evaluation was according to the US for ALNs in our center. If no ALNs were detected or had a cortical thickness <3 mm, cN0 was recorded, The abnormal ALN group were determined by one of the following three criteria: (i) Regular target annular lymph node with a peripheral cortical thickness ≥3 mm; (ii) eccentric target annular lymph node with a local cortical thickness ≥3 mm, and (iii) hypoechoic lymph node without a hilus structure of the lymph node; cN1 was recorded for these results.^5^ CNB or fine needle aspiration cytology (FNAC) was performed following cN1 result by US, pN1 was recorded following positive pathology by CNB or fine needle aspiration (FNA).^6^ For patients with negative pathology by CNB or FNA, and patients with cN0 by US, SLNB was then performed.^7^ If no metastasis was proven by histopathological interpretation from SLNB, then it was recorded as pN0, otherwise it was recorded as pN1. Thus, the information of cN0/cN1 as well as pN0/pN1 by both PE and US were obtained.

### Sentinel lymph node biopsy

The ^99m^Tc‐rituximab was used as the tracer for SLNB. The tracer was injected into two of the following sites: the peritumoral breast parenchyma, subcutaneous and subareolar tissues. The injection dose was 18.5 MBq, injected in the morning on the day of surgery, or 37 MBq on the day before surgery. Sentinel lymph node biopsy was performed under local anesthesia. SLNs were identified by a handled gamma detection probe (Neoprobe, USA or Crystal, Germany). All radioactive nodes with a counting rate ≥10% of the hottest node were removed. If no ALN metastasis was proven by histopathological interpretation from SLNB, then it was recorded as pN0, whereas it was recorded as pN1 when ALN metastasis was proven by histopathological interpretation from SLNB.^7^, ^8^

### Study endpoint and criteria

True positive (TP) was defined as positive FNA/CNB/SLNB findings or negative FNA/CNB findings followed by positive SLNB findings. True negative (TN) was defined as negative SLNB findings or negative FNA/CNB followed by negative SLNB findings. False negative (FN) was defined as cases with negative PE/US findings but subsequent positive SLNB findings. False positive (FP) was defined as cases with positive PE/US findings, but negative FNA/CNB/SLNB findings. The sensitivity (TP/[TP + FN]), specificity (TN/[TN + FP]), positive predictive value (PPV: TP/[TP + FP]), and negative predictive value (NPV: TN/[TN + FN]) were calculated. Accuracy is defined as the proportion of TP + TN in the population.

### Statistical analysis

Mean ± standard deviation was used for description of measurement data, and constituent ratio was applied for description of enumeration data. Pathology was regarded as the gold standard for analysis of sensitivity, specificity, accuracy, positive predictive value, and negative predictive value of ALN metastasis by PE and US, respectively. The difference of sensitivity, specificity and accuracy between PE and US was tested by McNemar chi‐square test. The standard curve was drawn and the area under the curve was calculated, and the differences under the standard curve between PE and US were compared. All statistical computations were performed using SPSS, version 22.0 (SPSS Inc., Chicago, IL).

## Results

**Figure 1:**
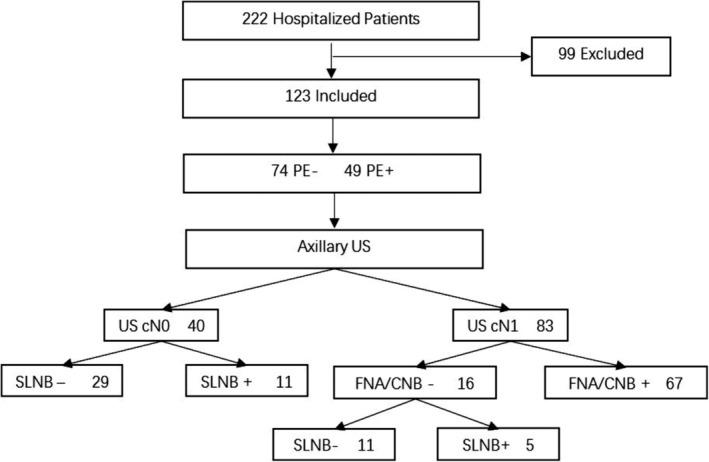
Flow diagram of study. Procedure of assessing ALN pathology.

A total of 222 consecutive and nonselective breast cancer patients were treated between September 2018 and November 2018 in the Breast Cancer Center of Peking University Cancer Hospital, from which 123 cases were finally included in this study according to the inclusion and exclusion criteria. The basic information of the included patients is shown in Table 1. The ALN evaluation flowchart for the included patients are shown in Fig 1.

**Table 1:** Patient characteristics in the study

		*n*	%
Age			
	20–29	2	1.6
	30–39	10	8.1
	40–49	29	23.6
	50–59	36	29.3
	60–69	44	35.8
	>70	2	1.6
T size (cm)			
	≤1.0	2	1.6
	1.1–2.0	44	35.8
	2.1–5.0	66	53.7
	>5.0	11	8.9
ER (%)			
	−	18	14.6
	+	105	85.4
PR (%)			
	−	27	22.0
	+	96	78.0
HER‐2			
	−	92	74.8
	+	30	24.4
	Unknown	1	0.8
PE			
	cN0	74	60.2
	cN1	49	39.8
US			
	cN0	40	32.5
	cN1	83	67.5
ALN pathology			
	pN0	40	32.5
	pN1	83	67.5

ALN, axillary lymph node; ER, estrogen receptor; HER‐2, human epidermal growth factor receptor 2; PE, physical examination; PR, progesterone receptor; T, tumour; US, ultrasound.

**Figure 2:**
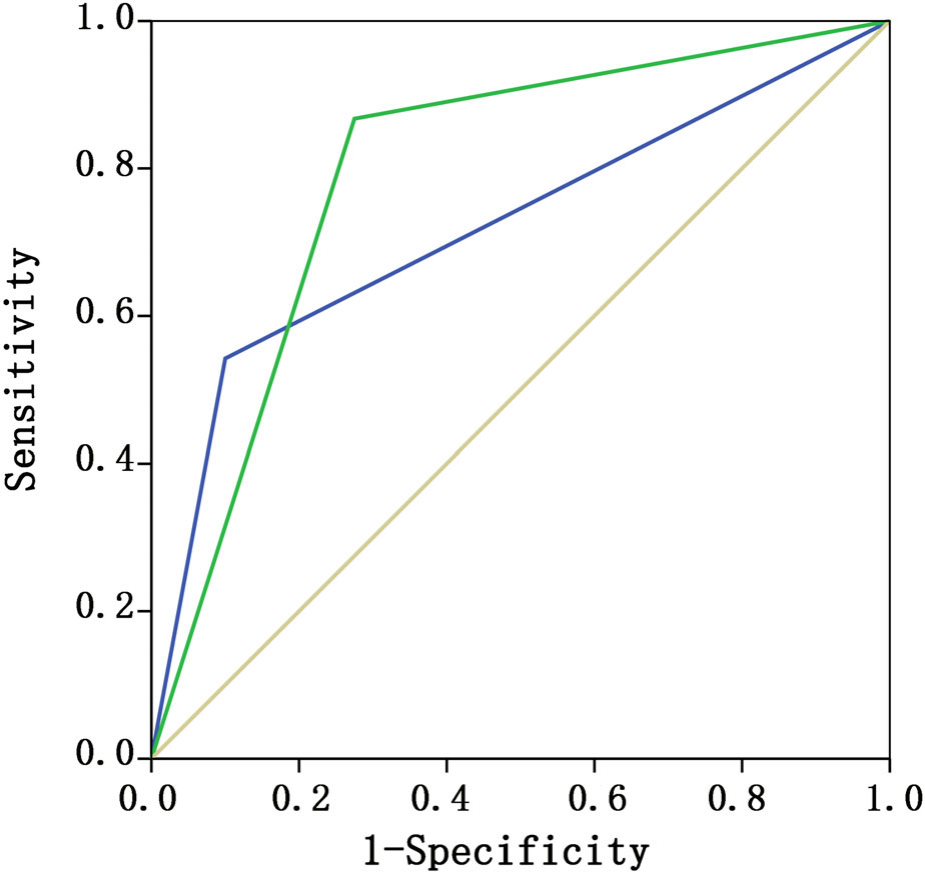
The area under the ROC curve by PE and US of all patients. ROC curves (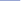) PE, (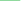) US and (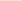) reference.

There were 74 ALN negative cases and 49 ALN positive cases by PE, respectively; 83 ALN negative cases and 40 ALN positive cases by US, respectively; 83 ALN negative cases and 40 ALN positive cases as confirmed by pathology, respectively (Table 2). Compared with pathological results, the sensitivity, specificity, accuracy, positive predictive value, and negative predictive value for PE were 54.2%, 90.0%, 65.9%, 91.8%, and 48.7%, respectively; and the sensitivity, specificity, accuracy, positive predictive value, and negative predictive value for US were 86.8%, 72.5%, 82.1%, 86.8%, and 72.5%, respectively. The area under the ROC curve of PE and US were 0.721 (95% CI, 0.630–0.812) and 0.796 (95% CI, 0.704–0.888), respectively (Fig 2).

**Table 2:** Physical examination, ultrasound and pathology results of ALNs of all patients

		Pathology results of ALNs		
		+	−	Total
PE	+	45	4	49
	−	38	36	74
US	+	72	11	83
	−	11	29	40
Total		83	40	123

PE, physical examination; US, ultrasound.

**Figure 3:**
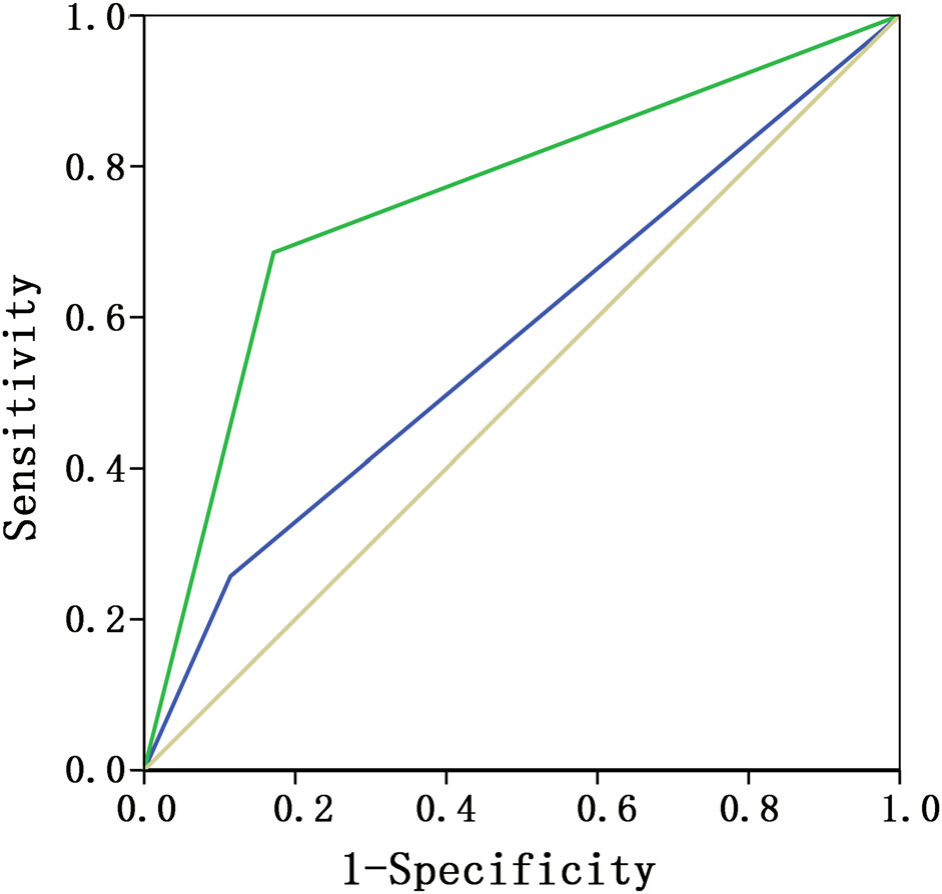
The area under the ROC curve by PE and US of patients whose ALN was diagnosed as regular target annular lymph node with a peripheral cortical thickness ≥3 mm or eccentric target annular lymph node with a local cortical thickness ≥3 mm. ROC curves (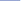) PE, (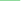) US and (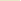) reference.

In the patients whose ALN was diagnosed as regular target annular with regular target annular lymph node with a peripheral cortical thickness ≥3 mm or eccentric target annular lymph node with a local cortical thickness ≥3 mm, 35 cases were confirmed to be ALN positive and 35 cases confirmed to be ALN negative by pathology (Table 3). In this group of patients, compared with pathological results, the sensitivity, specificity, accuracy, positive predictive value, and negative predictive value of PE versus US were 25.7% versus 68.6%, 88.6% versus 82.9%, 57.1% versus 75.7%, 69.2% versus 80.0%, and 54.3% versus 72.5%, respectively. The diagnostic accuracy of US was significantly higher than that of PE, *P* < 0.001. In this group of patients, the ROC curve of PE and US were 0.571 (95% CI, 0.437–0.706) and 0.757 (95% CI, 0.640–0.874), respectively (Fig 3).

**Table 3:** Physical examination, ultrasound and pathology results of ALNs of patients whose ALN was diagnosed as regular target annular lymph node with a peripheral cortical thickness ≥3 mm or eccentric target annular lymph node with a local cortical thickness ≥3 mm

		Pathology results of ALNs		
		+	−	Total
PE	+	9	4	13
	−	26	31	57
US	+	24	6	30
	−	11	29	40
Total		35	35	70

PE, physical examination; US, ultrasound.

Therefore, we believe that the sensitivity and accuracy of US in evaluating ALN is superior to that of PE, especially for ALN with cortical thickness >3 mm. Therefore, US should replace PE as a routine clinical staging method for ALN in breast cancer.

## Discussion

### Main findings of the study

Compared with pathological results, the sensitivity, specificity, accuracy, positive predictive value, and negative predictive value of PE vs US were 54.2% vs 86.8%, 90.0% vs 72.5%, 65.9% vs 82.1%, 91.8% vs 86.8%, and 48.7% vs 72.5%, respectively. The sensitivity and accuracy of US were higher than that of PE. For patients whose ALN was diagnosed as regular target annular lymph node with a peripheral cortical thickness ≥3 mm or eccentric target annular lymph node with a local cortical thickness ≥3 mm by US, compared with pathological results, the sensitivity, specificity, accuracy, positive predictive value, and negative predictive value of PE vs US were 25.7% vs 68.6%, 88.6% vs 82.9%, 57.1% vs 75.7%, 69.2% vs 80.0%, and 54.3% vs 72.5%, respectively, indicating that the diagnostic sensitivity and accuracy of US is much higher than that of PE.

### US is the only objective examination for ALN evaluation without compromising the advantage of PE

In US examination, the merits of PE remain obvious. Patients were placed in a supine, lateral or semi‐lateral position, with their arms raised to fully show the axilla at the examination site. The axilla was examined in multiple sections by the direct contact method. Anatomically, ALNs in the axilla are divided into three regions according to the position relationship between ALNs and the pectoralis minor muscle. (i) Region I ‐ armpit lymph nodes located below the outer lateral margin of pectoralis minor muscle; (ii) region II ‐ located in the deep side of pectoralis minor muscle and (iii) region III ‐ located between the medial edge of pectoralis minor muscle and the inferior clavicle. US was performed for the examination of ALNs in regions I, II and III, respectively. The number, size, shape, boundary, lymphatic hilum structure, echo of internal cortex and medulla, cortical thickness, vascular pattern and the relationship with surrounding soft tissue and blood vessels were observed. The ALNs were scanned by two vertical sections. The abnormal and typically enlarged lymph nodes were selected for measurement, with the longest diameter determined as the major axis, and the minor axis was measured along the section vertical to the major axis. Cortical thickness was measured for routine target annular lymph nodes. The US examination was completed by two US physicians,^5^, ^8^ making US an objective as well as a repeatable method. Compared with other organs, the breast is located on the body surface, and it is easy to implement PE and obtain more direct information. This is also the reason why for many years PE has been the primary, even the only, means of clinical staging of ALNs in breast cancer. At the same time, the breast and axilla are also among the most changeable anatomical areas in the human body with variable individual characteristics (race, age, reproductive status, menstrual status and habitus) in terms of shape, size and location, and are also two of the most unfixed organs. Because one of the features of the breast is its mobility, PE can fully and comprehensively examine ALNs in breast cancer patients, irrespective of the patients' body characteristics, whereas other objective examinations (CT/MRI/mammography, etc) would be unlikely to become standard methods for ALN evaluation for the same reason. However, PE is only a subjective examination method, and has inevitable inherent defects.^9^ Therefore, US is the only objective means to provide objective criteria while retaining the advantages of PE in adequately examining the axilla. The results of US in ALN evaluation are objective, repeatable and comparable, which is also convenient for academic research and communication.

### US is the basis for minimally invasive biopsy and accurate pathological staging of ALNs

Surgery is the earliest way to cure breast cancer and provide long‐term survival of patients.^10^ However, in the history of breast cancer treatment, the trauma caused by surgery is also obvious, and the trauma related to axillary dissection is particularly serious. With the concept establishment and significance determination of SLNB, a gradual increase in SLNB technology has allowed a considerable number of patients to avoid the trauma caused by axillary lymph node dissection (ALND). The examination of ALNs by US plays an important part in the reduction of trauma caused in the treatment of breast cancer. In our center, the US evaluation of the axilla is as follows: (i) If an ALN is hypoechoic under US, then US‐guided CNB is performed, or (ii) if a regular target annular lymph node with a peripheral cortical thickness ≥3 mm, or eccentric target annular lymph node with a local cortical thickness ≥3 mm, then FNAC is performed. Irrespective of the CNB or FNAC procedure, as long as the pathological result is positive, then pN1 is recorded. In contrast, if an ALN was suggested as negative or uncertain by US, or the pathology of US‐guided biopsy (CNB/FNB) was negative, then sentinel lymph node biopsy (SLNB) would be performed.^5^, ^6^, ^7^ It is not difficult to see that US has become the standard method for the clinical staging of ALN and the basis of ALN biopsy (CNB/FNB/SLNB) in breast cancer in our center.

### The dilemma of US replacing PE and limitation of the study

Theoretically, there is a general trend to replace the more subjective PE with an objective imaging method that retains the merits of PE, for clinical staging of ALNs in breast cancer and to guide precise pathological staging. Previous studies have shown that the sensitivity and specificity for PE was 25%–35.5% and 93%–98.4%, respectively.^9^, ^11^, ^12^ Our results showed that the sensitivity and specificity for PE was 54.2% and 90.0% respectively. Other studies have reported that the sensitivity and specificity for US was 54.7%–92.3%, and 80.4%–97.1% respectively.^2^ Our study showed a similar result of sensitivity and specificity for US, 86.8% and 72.5%, respectively. However, in reality and in the scientific literature, US is not generally applied in the clinical staging of ALNs, mainly for the following reasons: (i) The standard of US use in the evaluation of ALNs varies considerably among centers with no consensus drawn at present^3^, ^13^, ^14^, ^15^, ^16^, ^17^, ^18^ (ii) Due to the worldwide differences in medical technology, the establishment of US as an independent discipline has been globally delayed, with limited popularity. Even in high volume centers in developed countries such as the USA, there remains a lack of specialist US physicians and US examination is carried out by clinical doctors of various specialties, rather than by physicians who specialize in US. As a result, the technical level in this specialty remains uneven and experience very limited, and it is therefore difficult to take part in academic discussions in this regard. (iii) In countries such as Europe, Japan and China, where the US specialty was established relatively early and US assessment of ALNs carried out earlier, it is difficult to communicate, draw lessons from, recognize and promote US because of the different standards. (iv) In addition, the popularity of US technology and the establishment of diagnostic criteria are difficult due to the objective influence of race and habitus of patients, the inherent characteristics of high motility of breast and regional lymph nodes, as well as the influence of different ages, menstrual and reproductive status.

This study came from highly specialized breast cancer centers with specialist breast US physicians, and it is difficult to popularize our results which is another limitation of the study. However, with the technical promotion and academic exchange of US, we believe that the application of US in the clinical staging of ALNs in breast cancer patients will be further improved in the future.

## Disclosure

All the authors declare that there are no conflict of interests.
